# Self-efficacy and turnover intention among personal trainers in commercial fitness clubs: The mediating role of job burnout

**DOI:** 10.1371/journal.pone.0359775

**Published:** 2026-10-05

**Authors:** Jianping Li, Yihao Li, Weihong Wang, Zongwei Liu, Liwei Yuan

**Affiliations:** School of Physical Education and Health, Heze University, Heze, Shandong, China; International University - Vietnam National University Ho Chi Minh City, VIET NAM

## Abstract

Although personal trainers (PTs) play an important role in promoting physical activity and providing individualized exercise guidance, their occupational well-being and retention in commercial fitness clubs have received limited scholarly attention. This study examined the association between self-efficacy and turnover intention among PTs and investigated whether job burnout mediated this relationship. A cross-sectional survey was conducted among 565 fitness-club employees who were actively providing personal-training services, including trainers with concurrent supervisory or managerial responsibilities. Confirmatory factor analysis indicated that the measurement model demonstrated acceptable fit, and reliability and validity tests supported the adequacy of the measurement instruments. Correlation analyses showed that self-efficacy was negatively associated with job burnout and turnover intention, whereas job burnout was positively associated with turnover intention. Bootstrap mediation analyses further indicated that job burnout served as an indirect mechanism linking self-efficacy to turnover intention, with emotional exhaustion showing the most consistent mediating role. These findings suggest that self-efficacy may be an important psychological resource associated with lower burnout and weaker turnover intention among PTs. From a practical perspective, strategies aimed at strengthening PTs’ self-efficacy, preventing emotional exhaustion, and reducing burnout may help improve staff retention in commercial fitness clubs.

## Introduction

Regular physical activity is widely recognized as an essential contributor to public health and the prevention of chronic diseases [1]. China’s National Fitness Plan (2021–2025) emphasized public participation in physical activity and the development of fitness services [2]. In 2023, the total output of China’s sports industry reached RMB 3.6741 trillion, including RMB 276.0 billion generated by physical fitness and leisure activities [3]. An industry report summarized by the General Administration of Sport of China estimated that China had 69.75 million offline paying fitness members, 36,447 commercial fitness clubs, and 42,177 fitness studios in 2023 [4]. These figures highlight the substantial scale of the industry and the importance of maintaining a stable fitness-service workforce. Within this context, PTs provide exercise guidance and motivational support, and their professional competence and interpersonal skills are important to client relationships [5]. However, despite their important role in health promotion, PTs’ occupational well-being and retention in commercial fitness clubs remain insufficiently examined. Research in fitness organizations has identified interpersonal relationships, organizational communication, and emotional labor as relevant aspects of service work [6,7]. Using the Copenhagen Burnout Inventory, previous research involving 115 PTs found that 33.0% met the threshold for personal burnout, 29.6% for work-related burnout, and 17.4% for client-related burnout [8]. Although these findings were obtained outside China, they indicate that burnout represents a meaningful occupational risk among fitness professionals. Research on early-career PTs has emphasized the role of trainer–client relationships in client retention [9]. Disruption of these relationships is one reason to examine trainers’ occupational well-being and turnover intention.

Self-efficacy may be an important psychological factor associated with PTs’ burnout and turnover intention [10]. Self-efficacy refers to individuals’ beliefs in their ability to organize and execute the actions required to manage prospective situations [11]. In commercial fitness clubs, PTs’ self-efficacy may be reflected in their confidence in applying professional knowledge, designing and adjusting exercise programs, communicating effectively with clients, solving work-related problems, and coping with occupational challenges. PTs with stronger self-efficacy may be better able to manage demanding work situations, regulate negative emotions, and maintain a sense of professional competence. Among Chinese university teachers, self-efficacy moderated the association between work stress and burnout [12]. This finding supports examining self-efficacy as a personal resource, while distinguishing that moderating role from the mediation model examined here. Burnout is commonly characterized by emotional exhaustion, depersonalization, and reduced personal accomplishment [13]. Associations between burnout and turnover intention have been reported in nursing and teaching samples [14]. From a resource-based perspective, lower self-efficacy may impair PTs’ ability to cope with occupational demands, thereby increasing burnout and ultimately strengthening their intention to leave. Job burnout may therefore mediate the relationship between self-efficacy and turnover intention.

Although previous studies have examined burnout and turnover intention among nurses and teachers, evidence from commercial fitness settings remains limited. This occupational gap alone, however, is not the principal theoretical gap addressed here. Prior research has commonly modeled self-efficacy and burnout as global constructs or treated burnout as a single intervening mechanism. Such aggregation implicitly assumes that distinct efficacy beliefs are associated with turnover intention through interchangeable forms of strain. This assumption is theoretically restrictive. Within the social cognitive and job demands-resources perspectives developed below, efficacy beliefs concern particular capabilities, whereas the burnout dimensions represent different forms of strain. A global mediation estimate therefore cannot determine whether different efficacy resources converge on a common pathway or whether particular resources operate through conceptually aligned burnout dimensions. PTs provide a useful setting in which to examine this question because their work simultaneously requires technical problem solving, intensive interpersonal interaction, continuous adaptation, emotional labor, and commercial performance. The contribution is therefore a dimension-specific test of mediation, not the transfer of established relationships to a new occupational group. By estimating nine indirect pathways linking three self-efficacy dimensions to turnover intention through three burnout dimensions, the analysis distinguishes a convergent mechanism, in which different efficacy beliefs operate through the same burnout dimension, from a differentiated mechanism, in which specific efficacy domains operate through functionally aligned burnout dimensions. This design tests whether the self-efficacy-burnout-turnover association is uniform or comprises distinct pathways that an aggregate model could conceal.

The study accordingly makes two related theoretical contributions. First, it examines whether self-efficacy functions as a general personal resource across burnout dimensions or whether its indirect associations with turnover intention depend on the type of capability belief involved. Second, through exploratory dimension-specific mediation analyses, it assesses whether emotional exhaustion, depersonalization, and reduced personal accomplishment account for these indirect associations equivalently. These distinctions allow the study to refine, rather than merely reproduce, the proposed mediation mechanism. The PT context strengthens this examination because professional, relational, and adaptive demands are simultaneously salient in commercial fitness work. Practically, the findings may inform targeted retention strategies, including professional training and mentoring to strengthen self-efficacy, supportive workload and supervisory practices to reduce emotional exhaustion, and recognition and career-development systems to enhance professional accomplishment. Accordingly, this study addresses four research questions: (1) How are the three dimensions of self-efficacy associated with the three dimensions of job burnout? (2) How are the three dimensions of self-efficacy associated with turnover intention? (3) How are the three dimensions of job burnout associated with turnover intention? and (4) Which of the nine dimension-specific indirect paths from self-efficacy to turnover intention through burnout are supported? These questions guide the theoretical framework, hypotheses, analyses, and discussion.

## Literature review and hypotheses development

### Theoretical foundations and occupational context

PTs’ work involves professional exercise guidance and the maintenance of client relationships [5]. In fitness services, employees’ emotional expression during interactions with members has also been associated with rapport and word of mouth [7]. These professional and interpersonal responsibilities provide the occupational context for the present study. From a theoretical perspective, maintaining service relationships while responding to work demands may require sustained emotional effort. However, workload, sales pressure, scheduling, and compensation arrangements were not directly measured here. They are considered plausible contextual influences rather than established characteristics of every participant’s job.

The proposed relationships are grounded primarily in social cognitive theory, with job demands-resources theory used as a complementary interpretive framework. Social cognitive theory provides the explanatory basis for positioning self-efficacy as an antecedent: efficacy beliefs shape how individuals appraise challenging situations, regulate emotions, select coping strategies, and persist when difficulties arise [15]. It therefore informs the predicted associations of self-efficacy with burnout and turnover intention. Job demands-resources theory has a narrower role in the present study. It conceptualizes self-efficacy as a personal resource and burnout as a form of strain associated with resource depletion under demanding working conditions [16]. However, the study does not measure job demands, job resources, or resource-buffering interactions. It therefore does not test the full job demands-resources model or its health-impairment and motivational processes. The theory is used to organize the interpretation of burnout-related resource depletion, not to estimate the effects of unmeasured work characteristics. Thus, social cognitive theory explains why capability beliefs may shape appraisal and coping, whereas job demands-resources theory helps interpret why diminished personal resources may be expressed as burnout before withdrawal-related cognitions emerge.

### Self-efficacy and job burnout

Self-efficacy refers to an individual’s belief in their capacity to organize and execute the actions required to manage prospective situations and achieve desired outcomes. In occupational settings, it shapes employees’ appraisal of job demands, confidence in problem solving, persistence after setbacks, and ability to regulate negative emotions under pressure [11]. Employees with stronger self-efficacy are therefore generally better able to cope with occupational stress and preserve their psychological resources. In the present study, self-efficacy comprises three dimensions: cognition and development efficacy, interpersonal communication and collaboration efficacy, and learning and creative self-efficacy. Cognition and development efficacy reflects PTs’ confidence in understanding professional requirements, applying exercise-related knowledge, solving work problems, and supporting their professional growth. Interpersonal communication and collaboration efficacy reflects confidence in managing client relationships, communicating with colleagues, and resolving interpersonal difficulties. Learning and creative self-efficacy reflects confidence in acquiring new knowledge, adapting to changing client needs, and developing flexible training solutions. These forms of self-efficacy may protect PTs from the three dimensions of job burnout: emotional exhaustion, depersonalization, and reduced personal accomplishment. Emotional exhaustion refers to the depletion of emotional and psychological energy; depersonalization involves detached, indifferent, or cynical attitudes toward clients or work; and reduced personal accomplishment reflects a diminished sense of competence and professional effectiveness [13]. PTs with stronger cognition and development efficacy may feel more capable of managing technical demands and maintaining professional progress, thereby reducing feelings of overload and ineffectiveness. Stronger interpersonal communication and collaboration efficacy may help them manage client expectations, emotionally demanding interactions, and workplace relationships without becoming emotionally depleted or detached. Similarly, stronger learning and creative self-efficacy may enable PTs to respond flexibly to unexpected situations and changing client needs, supporting both emotional resilience and professional accomplishment. In contrast, PTs with lower self-efficacy may perceive occupational demands as less controllable, making them more vulnerable to emotional exhaustion, depersonalization, and reduced personal accomplishment. Therefore:

H1: The dimensions of self-efficacy are negatively associated with the dimensions of job burnout among PTs in commercial fitness clubs.

### Self-efficacy and turnover intention

Turnover intention refers to an employee’s conscious and deliberate willingness to leave their current employment and is widely regarded as a proximal cognitive precursor to actual turnover behavior [16]. From a social cognitive perspective, employees who believe they can manage occupational challenges are more likely to persist, maintain work engagement, and seek solutions to difficulties. By contrast, those who perceive themselves as unable to meet work demands may view withdrawal or resignation as a possible coping response [11]. For PTs, cognition and development efficacy may strengthen confidence in professional competence, career growth, and the ability to resolve work-related problems. Interpersonal communication and collaboration efficacy may help them maintain constructive relationships with clients, colleagues, and managers, thereby reducing interpersonal strain. Learning and creative self-efficacy may support adaptation to changing client preferences, emerging exercise knowledge, and evolving commercial expectations. These personal resources may increase PTs’ perceived control over their work and reduce their tendency to consider leaving. Conversely, PTs with lower self-efficacy may experience occupational demands as more difficult to manage, perceive fewer opportunities for successful adaptation, and become more likely to adopt withdrawal-related cognitions. Accordingly:

H2: The dimensions of self-efficacy are negatively associated with turnover intention among PTs in commercial fitness clubs.

### Job burnout and turnover intention

Job burnout represents a prolonged psychological response to chronic occupational stress and reflects the progressive depletion of employees’ emotional, interpersonal, and motivational resources [13]. Prospective studies have linked burnout with job dissatisfaction and sickness absence [17], and a meta-analysis has examined its association with impaired job performance [18]. Each burnout dimension may contribute to turnover intention through a distinct psychological process. Emotional exhaustion may increase thoughts of leaving because emotionally depleted PTs may find it increasingly difficult to sustain energy, enthusiasm, and client-centered service. This may be relevant to personal training because emotional expression and rapport are important in fitness-service interactions. Depersonalization may weaken PTs’ attachment to their work by encouraging detached or cynical attitudes toward clients, colleagues, or the organization. Because PTs’ work relies heavily on personalized trainer–client relationships, prolonged emotional detachment may reduce occupational involvement and commitment. Reduced personal accomplishment may similarly increase turnover intention by causing PTs to question their professional competence, effectiveness, and long-term suitability for the occupation. As these burnout-related experiences intensify, leaving the job may increasingly be perceived as a means of escaping an emotionally and psychologically unsustainable work environment. Therefore:

H3: The dimensions of job burnout are positively associated with turnover intention among PTs in commercial fitness clubs.

### The mediating role of job burnout

The preceding arguments suggest that job burnout may help explain the association between self-efficacy and turnover intention. Social cognitive theory provides the primary explanatory mechanism. PTs with stronger self-efficacy may appraise professional, interpersonal, and commercial challenges as more manageable, adopt more adaptive coping strategies, persist when difficulties arise, and regulate negative emotions more effectively [11]. Job demands-resources theory provides a complementary interpretive account: self-efficacy can be viewed as a personal resource, whereas burnout represents strain associated with resource depletion [15]. Because actual job demands and job resources are not included in the model, this study does not test whether demands produce burnout or whether self-efficacy buffers their effects. Within these limits, lower self-efficacy may be associated with greater emotional exhaustion, psychological detachment, or a reduced sense of personal accomplishment, which may in turn be associated with stronger turnover intention. The three burnout dimensions represent related but distinct candidate mechanisms. Emotional exhaustion may link lower self-efficacy with turnover intention through depleted emotional energy. Depersonalization may operate through weaker attachment to clients, colleagues, or the organization. Reduced personal accomplishment may operate through diminished perceptions of competence and professional value. These arguments support a family-level expectation of mediation. Prior theory and evidence are not sufficiently specific, however, to predict which of the nine possible combinations of self-efficacy and burnout dimensions should carry an indirect association. Accordingly:

H4: The dimensions of job burnout collectively account for indirect associations between the dimensions of self-efficacy and turnover intention among PTs in commercial fitness clubs.

H4 is a family-level hypothesis about the presence of mediation. The nine indirect paths formed by pairing each self-efficacy dimension with each burnout dimension are estimated as exploratory analyses because theory and prior evidence do not provide a sufficient basis for separate directional predictions for every pairing. These estimates are used to identify the pattern of mediation rather than to test nine distinct confirmatory subhypotheses. The proposed conceptual model is shown in Fig 1.

**Fig 1:**
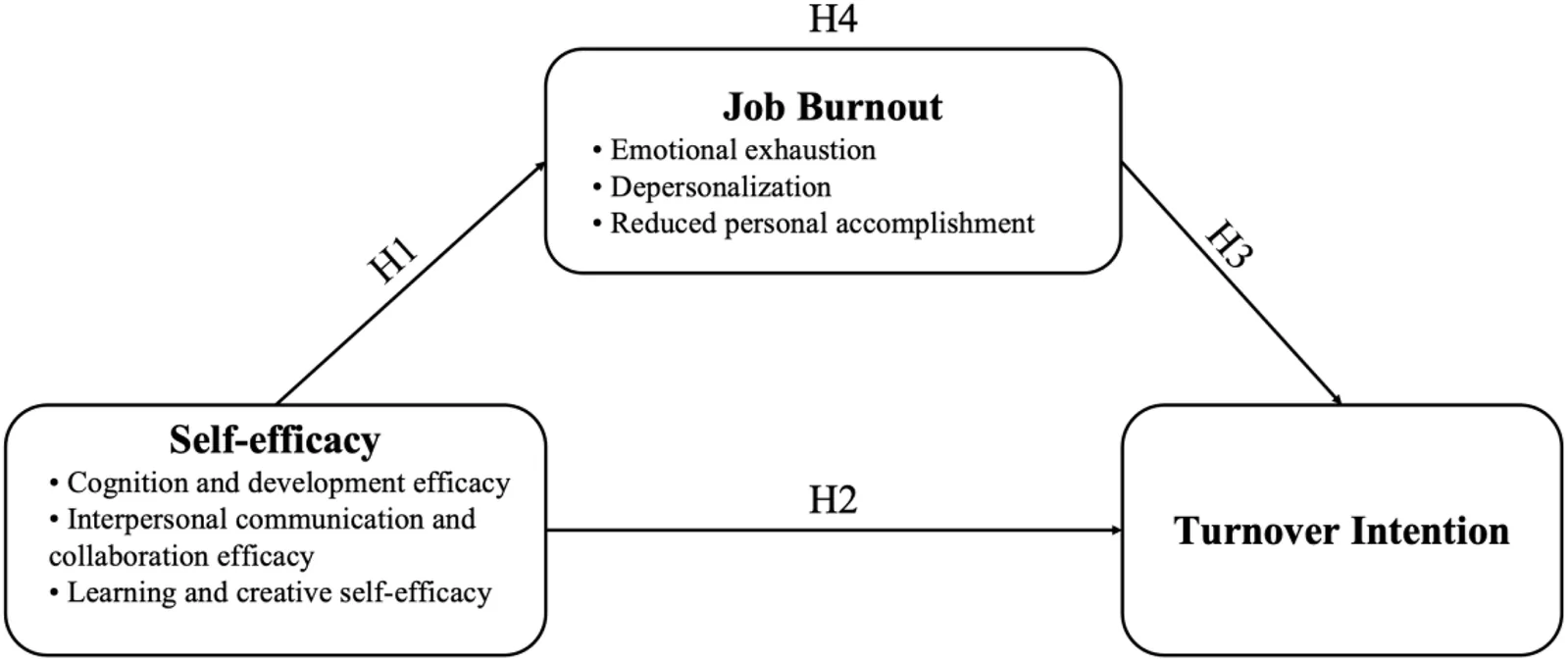
Proposed conceptual model of the relationships among self-efficacy, job burnout, and turnover intention among personal trainers in commercial fitness clubs.

## Methods

### Participants and procedures

This study used a cross-sectional survey design to examine self-efficacy, job burnout, and turnover intention among individuals actively providing personal-training services in commercial fitness clubs in Qingdao, Shandong Province, China. The study protocol was reviewed and approved by the Ethics Committee of Heze University on December 27, 2025, before the commencement of data collection (Approval No.: HZU-202601–077). All procedures involving human participants were conducted in accordance with the ethical standards of the institutional research committee and the principles of the Declaration of Helsinki. At the club level, a list of commercial fitness clubs operating in Qingdao was compiled through online searches. Fifteen clubs were randomly selected from this list and invited to participate. Nine clubs agreed to participate, five declined, and one did not respond. All nine participating clubs were branches of commercial fitness chains. Thus, although clubs were randomly selected for invitation from the compiled list, final participation depended on institutional consent. Within each participating club, eligible PTs who were present during the research team’s site visits were approached on site and invited to participate. Club managers facilitated access to potential participants but did not select individual respondents and were not involved in questionnaire completion, collection, or data processing. At the individual level, participants were eligible if they (a) were currently engaged in the direct provision of personal-training services to clients in a commercial fitness club, (b) were at least 18 years old, and (c) had at least one year of work experience in the fitness industry. Eligibility was determined according to participants’ actual personal-training duties rather than job title alone. Employees holding supervisory or managerial positions were therefore eligible if they continued to provide direct personal-training services. All 63 Coaching Supervisors and all 24 Coaching Managers included in the analytic sample continued to provide personal-training services in addition to their supervisory or managerial responsibilities.

Data were collected from January 1, 2026, to April 30, 2026. Before survey administration, members of the research team contacted managers or administrators of the participating clubs to explain the study purpose and procedures and obtain permission to approach eligible personnel. Questionnaires were distributed and collected by trained members of the research team. To reduce social desirability bias and protect participant privacy, respondents completed the questionnaires independently and returned them directly to the researchers. Before completing the survey, all participants received an information sheet explaining the study objectives, procedures, voluntary nature of participation, confidentiality of responses, and their right to withdraw at any time without penalty. Participants were informed that the questionnaire was anonymous, that no personally identifiable information would be collected, and that the data would be used only for academic research purposes. Written informed consent was obtained before questionnaire completion. No monetary or material compensation was provided. A total of 600 paper-based questionnaires were distributed and collected on site. During data screening, 35 questionnaires (5.8%) were excluded because they contained incomplete information. A questionnaire was classified as incomplete if any required item was unanswered. Questionnaires containing missing responses were excluded before statistical analysis, and no missing-value imputation was performed. No questionnaires were excluded because of duplication or other invalid-response criteria. Consequently, 565 complete questionnaires were retained for the final analysis, corresponding to an analyzable-questionnaire proportion of 94.2%. The demographic characteristics of the analytic sample are presented in Table 1. Respondents were nested within nine participating fitness clubs and may therefore have shared club-level working conditions. Club identifiers were not available in the analytic dataset used for the reported analyses. Accordingly, the primary structural equation model treated individual respondents as independent observations, and intraclass correlation coefficients, design effects, and cluster-adjusted standard errors were not estimated. The absence of a cluster-adjusted analysis should not be interpreted as evidence that within-club dependence was absent.

**Table 1:** Demographic characteristics (N = 565).

Variable	Category	N	%
Gender	Male	389	68.8
	Female	176	31.2
Age	18-25	163	28.8
	26-30	232	41.1
	31-35	114	20.2
	36-44	44	7.8
	45 years and above	12	2.1
Marital status	Unmarried	445	78.8
	Married	120	21.2
Education	Junior high school and below	29	5.1
	Senior high school or vocational school	125	22.1
	Junior college or university	391	69.2
	Postgraduate or above	20	3.5
Monthly personal-training hours	0-30	34	6.0
	31-60	56	9.9
	61-90	106	18.8
	91-120	198	35.0
	121-150	124	21.9
	151 and above	47	8.3
Monthly income level	0-5000 yuan	51	9.0
	5001-10000 yuan	142	25.1
	10001-15000 yuan	183	32.4
	15001-20000 yuan	119	21.1
	20001 ~ 30000 yuan	46	8.1
	30001 yuan and above	24	4.2
Position	Personal trainer	478	84.6
	Coaching Supervisor	63	11.2
	Coaching Manager	24	4.2
Years of experience	≤1 year	78	13.8
	>1 to ≤2 years	148	26.2
	>2 to ≤3 years	149	26.4
	>3 to ≤5 years	128	22.7
	>5 years	62	11.0

Note. Coaching supervisors and coaching managers were included only if they continued to provide direct personal-training services at the time of the survey.

### Sample-size planning

An a priori sample-size calculation was conducted using G*Power before formal data collection [19]. The calculation was based on a linear multiple regression model with three predictors. Assuming a small effect size (f^2^ = 0.02), a significance level of α = .05, and statistical power of  .80, the minimum required analytic sample was 550 participants. The planned questionnaire distribution was set at 600, exceeding the calculated minimum by 50 questionnaires. The final analytic sample comprised 565 participants and therefore exceeded the individual-level minimum indicated by the original calculation. However, the original calculation did not incorporate a design effect for potential within-club clustering. In addition, because the calculation was based on a regression model rather than on the nine dimension-specific indirect effects examined in the mediation analysis, it should be interpreted as a recruitment-planning criterion rather than evidence that each individual indirect-effect test achieved 80% statistical power.

### Measures

This study used a structured questionnaire to assess self-efficacy, job burnout, turnover intention, and demographic and work-related characteristics. All items were rated on a five-point Likert scale ranging from 1 (“strongly disagree”) to 5 (“strongly agree”), unless otherwise specified. Higher scores indicated higher levels of the corresponding construct. For scales originally developed in English, a translation-back-translation procedure was conducted to ensure conceptual equivalence between the English and Chinese versions. Before formal data collection, the questionnaire was reviewed by experts in sports management and pilot-tested with 50 PTs to evaluate item clarity, contextual relevance, and comprehensibility.

Self-efficacy was assessed using the 11-item Employee Self-Efficacy Scale developed by Zhang [20]. The original instrument was developed among new-generation employees in a manufacturing context and comprised three dimensions: cognition and development efficacy, interpersonal communication and collaboration efficacy, and learning and creative self-efficacy. To adapt the instrument to the PT context, occupation-general references in the original items were contextualized to personal-training work. Specifically, generic references to employees, work tasks, workplace interactions, and occupational development were reworded, where necessary, to refer to PTs’ professional duties, interactions with clients and colleagues, and work within commercial fitness clubs. The adaptation was intended to increase occupational relevance without altering the constructs being measured. The adapted items were included in the expert review and pilot test described above. Representative item content included confidence in applying professional knowledge and addressing work-related problems for cognition and development efficacy, confidence in communicating effectively with clients and collaborating with colleagues for interpersonal communication and collaboration efficacy, and confidence in learning new professional knowledge and developing flexible solutions to changing client needs for learning and creative self-efficacy. Higher scores indicated stronger self-efficacy.

Job burnout was assessed using the 22-item Fitness Personal Trainer Burnout Scale reported by Jia [21]. The instrument was adapted for the occupational context of fitness personal trainers and comprises nine emotional-exhaustion items, five depersonalization items, and eight personal-accomplishment items. The personal-accomplishment items were reverse-scored so that higher values represented greater reduced personal accomplishment and higher burnout.

Turnover intention was measured using the five-item Turnover Intention Scale attributed to Fan [22]. The scale assesses employees’ negative evaluations of continued employment and their intentions to leave their current organization, with higher scores indicating stronger turnover intention.

### Data analysis

Descriptive statistics and Pearson’s correlations were calculated to summarize the distributions and preliminary associations of the study variables. Before model estimation, the data were screened for missingness, potential outliers, and distributional characteristics. A questionnaire was excluded if any required item was unanswered. Accordingly, the analyses were based on complete questionnaires only, and no missing-value imputation was performed. Potential univariate outliers were screened using standardized scores, with absolute z-scores greater than 3.29 treated as potentially extreme. Multivariate outliers were additionally examined using Mahalanobis distance, with observations exceeding the chi-square criterion at p < .001 flagged for further inspection. Univariate distributional characteristics were evaluated using skewness and excess kurtosis. Measurement quality was evaluated using Cronbach’s alpha, composite reliability (CR), confirmatory factor analysis (CFA), average variance extracted (AVE), and discriminant validity. Cronbach’s alpha and CR values of 0.70 or above and AVE values of 0.50 or above were considered acceptable. Discriminant validity was assessed by comparing the square root of each construct’s AVE with its correlations with the other constructs [23].

Because all principal variables were collected from the same respondents in a single-wave self-report survey, potential common-method bias was assessed using complementary statistical procedures [24]. Harman’s unrotated single-factor test was first conducted using all 38 measurement items. A single-factor CFA was then estimated by constraining all 38 indicators to load on one common latent factor, and its fit was compared with that of the hypothesized seven-factor measurement model. In addition, full-collinearity variance inflation factors (VIFs) were calculated by regressing each of the seven construct scores on the remaining constructs. VIF values below 3.3 were interpreted as indicating the absence of serious full-collinearity concerns [25]. These procedures were treated as diagnostic checks rather than definitive evidence that common-method variance was absent.

The hypothesized relationships were tested using covariance-based structural equation modeling in AMOS 24 with maximum-likelihood estimation. Fit was assessed separately for the measurement and structural models using CMIN/DF, CFI, TLI, IFI, NFI, GFI, AGFI, and RMSEA. Model fit was considered acceptable when CMIN/DF was below 3.00, CFI, TLI, IFI, and NFI were approximately  .90 or higher, and RMSEA was below  .08; GFI and AGFI were reported as supplementary fit indices. Indirect effects were examined using bootstrapping and were considered statistically significant when their corresponding 95% confidence intervals did not include zero [26].

## Results

### Measurement model and preliminary analyses

Data screening indicated that no missing values remained in the final analytic dataset because questionnaires containing any unanswered required item had been excluded before analysis, and no missing-value imputation was performed. Examination of the seven construct scores showed no observations with absolute standardized scores greater than 3.29. Skewness values ranged from −0.381 to −0.041, and excess kurtosis values ranged from −1.115 to −0.856, indicating no severe univariate departures from normality. Mahalanobis-distance screening identified one observation exceeding the p < .001 criterion for seven variables. Inspection indicated that its values remained within the permissible ranges of the measures and provided no evidence of a data-entry error; the observation was therefore retained rather than removed solely on statistical grounds. These diagnostics supported the use of maximum-likelihood estimation while indicating that distributional assumptions should not be interpreted as being perfectly satisfied. Confirmatory factor analysis indicated that the measurement model demonstrated a good fit to the data: χ²/df = 1.835, CFI = .963, TLI = .960, RMSEA = .038, GFI = .900, AGFI = .884, IFI = .963, and NFI = .922. Descriptive statistics, reliability coefficients, full-collinearity VIF values, average variance extracted values, and inter-construct correlations are presented in Table 2. All Cronbach’s alpha and composite reliability values exceeded the recommended thresholds, indicating satisfactory internal consistency. The average variance extracted values met the recommended criterion, supporting convergent validity. In addition, the square root of the AVE for each construct exceeded its correlations with the other constructs, providing evidence of discriminant validity. All inter-construct correlations were statistically significant, and none exceeded  .80, suggesting that multicollinearity was unlikely to be a serious concern.

**Table 2:** Means, standard deviations, internal consistencies and correlations.

variables	Means	SD	α	CR	AVE	VIF	1	2	3	4	5	6	7
1. Cognition and development efficacy	3.585	1.028	0.919	0.919	0.739	1.236	**0.860**						
2. Interpersonal communication and collaboration efficacy	3.530	1.003	0.859	0.860	0.673	1.620	.311**	**0.820**					
3. Learning and creative self-efficacy	3.622	0.962	0.861	0.859	0.605	1.617	.272**	.545**	**0.778**				
4. Emotional exhaustion	3.599	0.808	0.933	0.933	0.607	1.929	−.285**	−.405**	−.394**	**0.779**			
5. Depersonalization	3.553	0.827	0.900	0.901	0.645	1.809	−.284**	−.383**	−.351**	.594**	**0.803**		
6. Reduced personal accomplishment	3.547	0.810	0.924	0.925	0.605	1.750	−.239**	−.359**	−.381**	.568**	.558**	**0.778**	
7. Turnover Intention	3.291	0.943	0.905	0.905	0.656	1.826	−.406**	−.476**	−.491**	.516**	.463**	.474**	**0.810**

** p < 0.01; *** p < 0.001. SD: Standard deviation. CR: Composite Reliability. Diagonal elements (in bold) are the square root of the average variance extracted (AVE).

Potential common method bias was assessed using Harman’s unrotated single-factor test, a single-factor CFA, and full-collinearity VIFs. Harman’s analysis extracted seven factors with eigenvalues greater than 1, and the first factor accounted for 37.53% of the total variance, indicating that no single factor explained the majority of the variance. The single-factor CFA showed poor fit to the data (χ² = 7179.03, df = 665, χ²/df = 10.796, CFI = .551, TLI = .525, IFI = .552, NFI = .528, GFI = .474, AGFI = .414, and RMSEA = .132). In contrast, the hypothesized seven-factor measurement model demonstrated substantially better fit (χ² = 1181.63, df = 644, χ²/df = 1.835, CFI = .963, TLI = .960, IFI = .963, NFI = .922, GFI = .900, AGFI = .884, and RMSEA = .038). The substantially poorer fit of the single-factor model indicates that the covariance among the measurement items was not adequately represented by a single latent factor, reducing concern that a single common method factor accounted for the observed relationships. The full-collinearity VIF values ranged from 1.236 to 1.929 and were all below the threshold of 3.30. Taken together, these diagnostic results suggest that common method variance was unlikely to pose a serious threat to the principal findings. Nevertheless, it cannot be completely ruled out because all variables were measured using self-reported data collected from the same respondents at a single time point.

### Structural model

The structural model was tested to examine the hypothesized direct relationships. The model demonstrated acceptable-to-good fit to the data: CMIN/DF = 2.278, CFI = .943, TLI = .938, IFI = .943, NFI = .903, GFI = .873, AGFI = .855, and RMSEA = .048. Although GFI and AGFI were below  .90, the incremental fit indices exceeded  .90 and RMSEA was below  .05, providing overall support for the adequacy of the structural specification. As shown in Fig 2 and Table 3, the three dimensions of self-efficacy were negatively associated with the three dimensions of job burnout. Cognition and development efficacy was negatively associated with emotional exhaustion (β = −0.140, p < .01), depersonalization (β = −0.156, p < .001), and reduced personal accomplishment (β = −0.102, p < .05). Interpersonal communication and collaboration efficacy was negatively associated with emotional exhaustion (β = −0.209, p < .001), depersonalization (β = −0.231, p < .001), and reduced personal accomplishment (β = −0.168, p < .01). Learning and creative self-efficacy was negatively associated with emotional exhaustion (β = −0.328, p < .001), depersonalization (β = −0.271, p < .001), and reduced personal accomplishment (β = −0.347, p < .001). These results supported H1.

**Fig 2:**
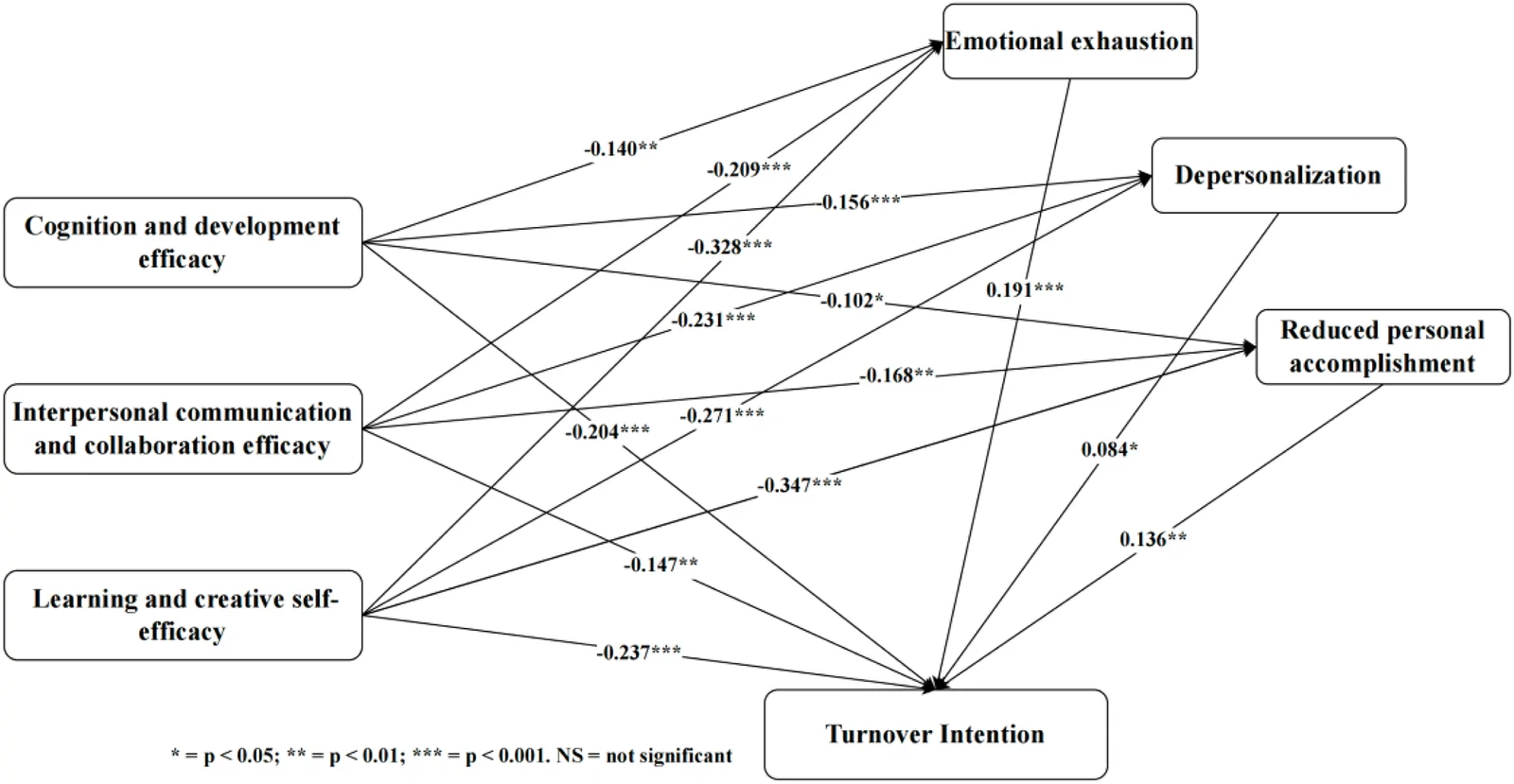
Empirical model.

**Table 3:** Hypothesis test results.

Structural path	β	S.E.	C.R.	Result
Cognition and development efficacy → emotional exhaustion	β1a=-0.140**	0.037	−3.258	Supported
Cognition and development efficacy → Depersonalization	β1a=-0.156***	0.038	−3.510	Supported
Cognition and development efficacy → Reduced personal accomplishment	β1a=-0.102*	0.036	−2.313	Supported
Interpersonal communication and collaboration efficacy → emotional exhaustion	β1a=-0.209***	0.053	−3.529	Supported
Interpersonal communication and collaboration efficacy → Depersonalization	β1a=-0.231***	0.054	−3.756	Supported
Interpersonal communication and collaboration efficacy → Reduced personal accomplishment	β1a=-0.168**	0.052	−2.763	Supported
Learning and creative self-efficacy → emotional exhaustion	β1a=-0.328***	0.052	−5.554	Supported
Learning and creative self-efficacy → Depersonalization	β1a=-0.271***	0.053	−4.483	Supported
Learning and creative self-efficacy → Reduced personal accomplishment	β1a=-0.347***	0.052	−5.667	Supported
Cognition and development efficacy → Turnover Intention	β1a=-0.204***	0.035	−5.119	Supported
Interpersonal communication and collaboration efficacy →Turnover Intention	β1a=-0.147**	0.049	−2.702	Supported
Learning and creative self-efficacy →Turnover Intention	β1a=-0.237***	0.052	−4.061	Supported
emotional exhaustion →Turnover Intention	β1a=0.191***	0.044	4.411	Supported
Depersonalization →Turnover Intention	β1a=0.084*	0.043	1.980	Supported
Reduced personal accomplishment →Turnover Intention	β1a=0.136**	0.044	3.262	Supported

* p < .05, **p < .01, ***p < .001; The dimension-specific path results provided overall support for H1, H2, and H3.

The three dimensions of self-efficacy were also negatively associated with turnover intention. Cognition and development efficacy (β = −0.204, p < .001), interpersonal communication and collaboration efficacy (β = −0.147, p < .01), and learning and creative self-efficacy (β = −0.237, p < .001) were all significantly associated with lower turnover intention. These results supported H2.

The three dimensions of job burnout were positively associated with turnover intention. Emotional exhaustion (β = 0.191, p < .001), depersonalization (β = 0.084, p < .05), and reduced personal accomplishment (β = 0.136, p < .01) were all significant predictors of turnover intention. These results supported H3.

### Mediation analysis

Bootstrap analysis was conducted to examine the indirect effects of the three self-efficacy dimensions on turnover intention through the three dimensions of job burnout. An indirect effect was considered statistically significant when its 95% confidence interval did not include zero. The results are presented in Table 4.

**Table 4:** Mediating effect test.

Indirect path	Indirect effect	S.E.	Lower	Upper	p
Cognition and development efficacy→ Emotional exhaustion→ Turnover Intention	−0.023	0.010	−0.047	−0.008	*
Interpersonal communication and collaboration efficacy→ Emotional exhaustion→ Turnover Intention	−0.036	0.042	−0.093	−0.004	*
Learning and creative self-efficacy→ Emotional exhaustion→ Turnover Intention	−0.056	0.036	−0.115	−0.020	*
Cognition and development efficacy→ Depersonalization→ Turnover Intention	−0.012	0.008	−0.031	0.000	0.057
Interpersonal communication and collaboration efficacy→ Depersonalization→ Turnover Intention	−0.018	0.041	−0.063	0.001	0.066
Learning and creative self-efficacy→ Depersonalization→ Turnover Intention	−0.021	0.036	−0.063	0.001	0.067
Cognition and development efficacy→ Reduced personal accomplishment→ Turnover Intention	−0.012	0.008	−0.033	−0.001	*
Interpersonal communication and collaboration efficacy→ Reduced personal accomplishment→ Turnover Intention	−0.021	0.025	−0.064	0.000	0.053
Learning and creative self-efficacy→ Reduced personal accomplishment→ Turnover Intention	−0.042	0.026	−0.093	−0.011	*

* p < .05, **p < .01, ***p < .001

Emotional exhaustion significantly mediated the relationships between each self-efficacy dimension and turnover intention. The indirect effect was significant for cognition and development efficacy, indirect effect = −0.023, 95% CI [−0.047, −0.008]; interpersonal communication and collaboration efficacy, indirect effect = −0.036, 95% CI [−0.093, −0.004]; and learning and creative self-efficacy, indirect effect = −0.056, 95% CI [−0.115, −0.020]. The indirect effects through depersonalization were not statistically significant. The confidence intervals included zero for cognition and development efficacy, indirect effect = −0.012, 95% CI [−0.031, 0.000], p = .057; interpersonal communication and collaboration efficacy, indirect effect = −0.018, 95% CI [−0.063, 0.001], p = .066; and learning and creative self-efficacy, indirect effect = −0.021, 95% CI [−0.063, 0.001], p = .067. Reduced personal accomplishment significantly mediated the relationships of cognition and development efficacy, indirect effect = −0.012, 95% CI [−0.033, −0.001], and learning and creative self-efficacy, indirect effect = −0.042, 95% CI [−0.093, −0.011], with turnover intention. Its indirect effect in the relationship between interpersonal communication and collaboration efficacy and turnover intention was not statistically significant, indirect effect = −0.021, 95% CI [−0.064, 0.000], p = .053. Taken together, these findings provided partial support for the family-level mediation expectation in H4. Because no separate hypotheses were proposed for the nine pairings, the path-specific pattern is interpreted as exploratory evidence.

## Discussion

All three dimensions of self-efficacy were negatively associated with emotional exhaustion, depersonalization, and reduced personal accomplishment. This finding is broadly consistent with research among medical staff in which self-efficacy mediated the association between sports involvement and job burnout [27]. Related research among Chinese university teachers examined self-efficacy as a moderator of the association between work stress and burnout [12]. These studies examine different roles for self-efficacy and provide complementary comparisons with the present model. The present study extends this evidence to PTs, whose occupational demands differ from those faced by teachers, healthcare professionals, and conventional service employees. PTs must integrate exercise instruction with sustained client interaction, emotional service, irregular schedules, and commercial performance expectations. Under these conditions, cognition and development efficacy may help PTs interpret technical and professional demands as manageable. Interpersonal communication and collaboration efficacy may support the management of difficult client interactions and workplace relationships, whereas learning and creative self-efficacy may facilitate adaptation to changing client needs and unexpected training situations. These capabilities may enable PTs to preserve emotional energy and maintain confidence in their professional effectiveness. The dimension-specific results also provide additional insight. Learning and creative self-efficacy showed comparatively strong negative associations with emotional exhaustion and reduced personal accomplishment. One possible explanation is that PTs routinely encounter clients with different physical conditions, preferences, motivation levels, and responses to exercise. Confidence in acquiring new knowledge and developing flexible training solutions may therefore be especially important for reducing repeated frustration and maintaining a sense of professional competence. Nevertheless, because the study was cross-sectional, it is also possible that PTs who already experience burnout evaluate their learning and adaptive capabilities less favorably. Longitudinal research is needed to clarify the temporal direction of these relationships. The findings are also consistent with occupation-specific evidence that burnout represents a meaningful concern among fitness professionals. However, previous research involving PTs has primarily documented the presence or correlates of burnout. The current study advances this literature by identifying specific psychological resources associated with each burnout dimension. At the same time, self-efficacy should not be interpreted as a substitute for supportive working conditions. Recent qualitative research in fitness franchises identified internal communication, interpersonal relationships, organizational climate, work-related stress, and work–life balance as important influences on employee satisfaction and well-being [6]. Therefore, although stronger self-efficacy may help PTs cope with demanding work, persistent workload, sales pressure, inadequate managerial support, or unstable compensation systems may exceed what individual psychological resources can reasonably offset.

The three dimensions of self-efficacy were negatively associated with turnover intention. This result is consistent with evidence linking self-efficacy to lower turnover intention among Lebanese nurses [10]. However, this association may depend on organizational conditions: a study of manufacturing employees found that higher self-efficacy was associated with greater turnover intention when organizational trust was low [28]. Employees who believe that they can solve work-related problems and adapt to occupational demands may perceive greater control over their work and may be less likely to view leaving as their primary coping option. The current findings add to previous research by showing that multiple forms of work-related self-efficacy are independently associated with turnover intention among PTs. Cognition and development efficacy may strengthen confidence in professional growth and future career prospects. Interpersonal communication and collaboration efficacy may reduce relationship-based strain involving clients, colleagues, and managers. Learning and creative self-efficacy may help PTs respond flexibly to changing client expectations and industry practices. Learning and creative self-efficacy demonstrated the strongest negative association with turnover intention among the three dimensions. This may reflect the dynamic nature of personal training, in which trainers must continually update their knowledge, adapt exercise programs, and respond to diverse client needs. PTs who perceive themselves as capable of learning and adapting may see more opportunities to resolve occupational difficulties without leaving their current employment.

Emotional exhaustion, depersonalization, and reduced personal accomplishment were positively associated with turnover intention. This finding is consistent with evidence linking burnout to turnover intention among nurses and to professional turnover intention among Chinese English language teachers [10,14,29]. Emotional exhaustion showed the largest standardized association with turnover intention among the three burnout dimensions. This pattern is theoretically plausible because emotional exhaustion reflects the direct depletion of energy required to perform the central tasks of personal training. PTs must repeatedly display enthusiasm, provide motivation, manage client emotions, and sustain individualized attention. When this emotional energy is depleted, continued participation in such intensive interpersonal work may become increasingly difficult, making departure appear to be a means of protecting remaining resources. Depersonalization and reduced personal accomplishment were also positively associated with turnover intention, but their associations were weaker. Depersonalization may weaken PTs’ emotional connection with clients and the organization, whereas reduced personal accomplishment may cause them to question their professional effectiveness and future suitability for the occupation. These results are consistent with the multidimensional conceptualization of burnout but also demonstrate that its components may have different occupational consequences.

Job burnout partially mediated the relationships between self-efficacy and turnover intention, but the indirect effects differed across burnout dimensions. This result is consistent with prior nursing research examining burnout as a mediator between self-efficacy and turnover intention, and between professional identity or occupational stress and turnover intention [10,29]. Rather than indicating a single, undifferentiated mediation process, the exploratory path estimates revealed a structured asymmetry. Emotional exhaustion was the only mediator shared by all three self-efficacy dimensions. Reduced personal accomplishment mediated the relationships involving cognition and development efficacy and learning and creative self-efficacy, whereas depersonalization did not significantly mediate any of the three relationships. This pattern shows what the dimension-specific analysis adds: it identifies which components of the conventional global chain carry the association and which do not. The convergence of all three self-efficacy dimensions on emotional exhaustion is compatible with the resource-depletion logic of job demands-resources theory. It is not, however, a test of the theory’s health-impairment process because actual job demands were not measured. From a social cognitive perspective, PTs with lower confidence in their professional, interpersonal, or adaptive capabilities may appraise occupational challenges as more difficult to control and expend greater emotional and cognitive effort in managing them. Emotional exhaustion may then be associated with stronger withdrawal-related cognitions. Reduced personal accomplishment showed a narrower pathway. Cognition and development efficacy and learning and creative self-efficacy concern professional competence, growth, task mastery, and adaptation. Their indirect associations through reduced personal accomplishment suggest a possible domain-matching explanation. Interpersonal communication and collaboration efficacy did not show this indirect path, possibly because confidence in relationship management is less directly involved in evaluations of technical achievement. The nonsignificant indirect paths through depersonalization indicate that the self-efficacy-turnover associations were not transmitted through psychological distancing in this sample. Because the nine specific paths were exploratory, these patterns should be treated as hypothesis-generating and tested in future confirmatory research.

This study has several theoretical and practical implications. Social cognitive theory is the primary explanatory framework: it links capability beliefs to appraisal, coping, persistence, and emotion regulation, thereby providing the basis for the proposed associations of self-efficacy with burnout and turnover intention. Job demands-resources theory is used more modestly as an interpretive lens for viewing self-efficacy as a personal resource and burnout as strain. The findings neither show that job demands caused burnout nor test whether self-efficacy buffered the effects of job demands. Accordingly, the study does not claim to extend the full job demands-resources model. Its theoretical contribution instead lies in decomposing self-efficacy and burnout to show that the indirect associations are not uniform. The exploratory results suggest a broad pathway through emotional exhaustion and a selective pathway through reduced personal accomplishment, whereas depersonalization did not account for the indirect associations. Dimension-specific mediation therefore reveals whether a candidate mechanism is shared across personal resources, limited to particular dimension pairings, or unsupported, information that an aggregate indirect effect cannot provide. Practically, commercial fitness clubs should combine individual-level development with improvements in working conditions. Professional training, mentoring, supervised practice, and constructive feedback may strengthen PTs’ professional, interpersonal, and adaptive self-efficacy. Managers should also reduce emotional exhaustion by monitoring workloads, limiting excessive consecutive sessions, ensuring adequate recovery time, distributing sales and client-service responsibilities fairly, and avoiding unrealistic performance targets. Recognition systems, transparent career pathways, developmental feedback, and continuing education may further support PTs’ sense of professional accomplishment. Burnout should not be treated solely as an individual problem. Qualitative evidence from fitness franchises highlights internal communication, interpersonal relationships, and organizational climate as relevant to employee satisfaction [6]. These themes may inform the development of retention strategies, although the effects of specific organizational interventions on actual retention require direct evaluation.

Several limitations should be acknowledged. First, the cross-sectional design precludes causal or temporal conclusions. Although the proposed model was theoretically grounded, reverse and reciprocal relationships remain possible. Burnout may weaken self-efficacy, and turnover intention may influence employees’ evaluations of their work experiences. Longitudinal, time-lagged, or experimental studies are needed to examine these possibilities. Second, participants were recruited through convenience sampling from commercial fitness clubs in Qingdao, Shandong Province. The findings may not generalize to PTs in other regions, independent studios, public fitness facilities, online training settings, or different cultural and labor-market contexts. Future studies should use broader, multi-region samples and consider multilevel differences among clubs. Third, all principal variables were measured using self-report questionnaires in a single survey. Although statistical procedures were used to assess common method bias, such procedures cannot eliminate concerns regarding response tendencies or shared measurement context. Future research could combine self-reports with supervisor assessments, client feedback, scheduling records, performance indicators, sickness absence, and actual turnover data. Fourth, the study did not directly measure job demands or job resources. The findings therefore cannot test the health-impairment, motivational, or buffering propositions of job demands-resources theory. Future models should include demands such as workload, sales pressure, emotional labor, and irregular schedules, together with resources such as autonomy, managerial support, and recovery opportunities. Fifth, the nine dimension-specific indirect paths were exploratory. Their apparent convergence and domain-matching patterns require replication using preregistered, adequately powered confirmatory studies. An additional limitation concerns the hierarchical structure of the data. Participants were recruited from nine commercial fitness clubs and may therefore have shared club-level working conditions, management practices, or organizational climates. Because club identifiers were not retained in the final analytic dataset, within-club dependence could not be quantified using intraclass correlations or design effects, and the primary SEM did not incorporate a multilevel or cluster-adjusted estimator. If responses were positively correlated within clubs, the reported standard errors and confidence intervals may be narrower than they would be under an analysis that explicitly accounted for clustering. Future studies should retain non-identifying club codes, recruit a larger number of clubs, and prespecify multilevel or small-cluster-appropriate analyses.

## Conclusion

This study examined the relationships among multidimensional self-efficacy, job burnout, and turnover intention among personal trainers working in commercial fitness clubs. The findings showed that the three dimensions of self-efficacy were negatively associated with burnout and turnover intention, whereas emotional exhaustion, depersonalization, and reduced personal accomplishment were positively associated with turnover intention. The exploratory dimension-specific indirect effects were not uniform: emotional exhaustion mediated the relationships between all three self-efficacy dimensions and turnover intention, reduced personal accomplishment showed selective mediation, and depersonalization did not show significant mediation. This asymmetry suggests that an aggregate burnout mechanism may conceal distinct pathways, but the path-specific interpretation requires confirmatory replication. Social cognitive theory provides the primary explanation for the role of efficacy beliefs, whereas job demands-resources theory offers a complementary resource-depletion interpretation. Because job demands and job resources were not measured, the findings should not be interpreted as a test or extension of the full job demands-resources model. Commercial fitness clubs should combine strategies that strengthen PTs’ professional, interpersonal, and adaptive self-efficacy with organizational measures aimed at reducing emotional exhaustion, supporting professional accomplishment, and improving working conditions. Given the cross-sectional and self-reported nature of the data, the findings should be interpreted as associations, and future longitudinal and multi-source research is needed to examine their temporal and causal relationships.

### Use of AI-assisted tools

During revision, OpenAI Codex was used to assist with English-language editing, drafting responses to reviewers, literature searching, identification and checking of candidate references, and generating and executing code. All AI-assisted wording, references, bibliographic information, and supplementary calculations were manually reviewed and verified by the authors against the original sources and/or underlying data before inclusion. Responsibility for the accuracy of the manuscript, references, analyses, and final interpretations rests entirely with the authors.

## Supporting information

S1 Table Item wording, adaptation/scoring information, and item-level factor loadings of the study measures. (DOCX)

S1 File The raw data of this study. (XLSX)
