# Supplementary material for: Self-efficacy and turnover intention among personal trainers in commercial fitness clubs: The mediating role of job burnout
Source: PLoS One. 2026 Oct 5;21(10):e0359775. doi: 10.1371/journal.pone.0359775 (PMC13637870; doi:10.1371/journal.pone.0359775)
Supplement: Supplementary file 1 [file pone.0359775.s001.docx]

**Supplementary Table S1**

Supplementary Table S1. Item wording, adaptation/scoring information, and item-level factor loadings of the study measures

Note. The English wording represents translations of the Chinese questionnaire items. SE = self-efficacy; JB = job burnout; TI = turnover intention. For the self-efficacy scale, selected occupational referents were contextualized to personal-training work without adding or deleting items. The burnout measure was the personal-trainer-specific version adapted by Jia from the MBI-GS. JB15–JB22 were reverse-scored so that higher values indicated greater reduced personal accomplishment.

**A. Self-efficacy: 11 items**

| **Dimension** | **Code** | **English item wording** | **Adaptation information** | **Loading** |
| --- | --- | --- | --- | --- |
| Cognition and development efficacy | SE1 | I feel that doing this job is the right choice and is meaningful. | Occupational referent contextualized to current PT work | 0.826 |
| Cognition and development efficacy | SE2 | When faced with newly assigned tasks or unexpected events, I am always confident. | Work-task context retained/adapted to PT work | 0.869 |
| Cognition and development efficacy | SE3 | I believe that I can bring substantial value to my team and organization. | Team/organizational referent contextualized to PT workplace | 0.896 |
| Cognition and development efficacy | SE4 | I am satisfied with my current job and enthusiastic about it. | Current-work referent contextualized to PT work | 0.846 |
| Interpersonal communication and collaboration efficacy | SE5 | I can communicate proactively with clients, regardless of whether I know them well. | Client referent contextualized to personal-training interactions | 0.861 |
| Interpersonal communication and collaboration efficacy | SE6 | I get along well with my colleagues and work collaboratively with them to complete the tasks at hand. | Colleague/teamwork context | 0.756 |
| Interpersonal communication and collaboration efficacy | SE7 | I am in good psychological condition and can quickly adjust my mindset when work-related problems arise. | Work-related self-regulation context | 0.840 |
| Learning and creative self-efficacy | SE8 | I am willing to explore new methods, techniques, or tools for my work. | Work context contextualized to PT practice | 0.824 |
| Learning and creative self-efficacy | SE9 | When faced with difficulties or problems, I can usually find new ways to address them. | No substantive construct change | 0.726 |
| Learning and creative self-efficacy | SE10 | I usually have multiple ways to solve a problem. | No substantive construct change | 0.747 |
| Learning and creative self-efficacy | SE11 | I am confident that I can handle unexpected situations using my own abilities. | No substantive construct change | 0.810 |

**B. Job burnout**

| Dimension | Code | English item wording | Adaptation/scoring information | Loading |
| --- | --- | --- | --- | --- |
| Emotional exhaustion | JB1 | Fitness instruction work makes me feel frustrated. | PT-specific wording | 0.766 |
| Emotional exhaustion | JB2 | I feel that my fitness instruction work is not understood by others. | PT-specific wording | 0.774 |
| Emotional exhaustion | JB3 | Fitness instruction work leaves me exhausted. | PT-specific wording | 0.768 |
| Emotional exhaustion | JB4 | I believe that I work very hard. | PT-specific version | 0.787 |
| Emotional exhaustion | JB5 | Fitness instruction work makes me feel overwhelmed and unable to cope. | PT-specific wording | 0.799 |
| Emotional exhaustion | JB6 | I feel that I will gradually lose patience with fitness instruction work. | PT-specific wording | 0.799 |
| Emotional exhaustion | JB7 | I feel that the methods I use in fitness instruction are inappropriate. | PT-specific wording | 0.766 |
| Emotional exhaustion | JB8 | My colleagues blame me for the problems they encounter. | Workplace referent | 0.785 |
| Emotional exhaustion | JB9 | I often look forward to having time off. | PT-specific version | 0.764 |
| Depersonalization | JB10 | Since working in fitness instruction, I have become more indifferent toward other people. | PT-specific wording | 0.821 |
| Depersonalization | JB11 | I am indifferent to what happens to other classmates.† | Verify administered wording | 0.812 |
| Depersonalization | JB12 | Fitness instruction work makes me feel discouraged. | PT-specific wording | 0.773 |
| Depersonalization | JB13 | I feel that I need to take a break for a period of time. | PT-specific version | 0.796 |
| Depersonalization | JB14 | When facing members of the public, I experience considerable pressure.† | Verify administered PT-specific referent | 0.812 |
| Reduced personal accomplishment | JB15 | I feel that my efforts will be rewarded. | Reverse-scored | 0.767 |
| Reduced personal accomplishment | JB16 | I believe that my work is valuable. | Reverse-scored | 0.770 |
| Reduced personal accomplishment | JB17 | I believe that fitness instruction work is meaningful. | Reverse-scored; PT-specific wording | 0.755 |
| Reduced personal accomplishment | JB18 | I gain a sense of satisfaction from fitness instruction work. | Reverse-scored; PT-specific wording | 0.772 |
| Reduced personal accomplishment | JB19 | I have my own goals in fitness work. | Reverse-scored; PT-specific wording | 0.778 |
| Reduced personal accomplishment | JB20 | I can carry out fitness instruction work with abundant energy. | Reverse-scored; PT-specific wording | 0.809 |
| Reduced personal accomplishment | JB21 | I am willing to learn new knowledge through fitness instruction work and improve myself.† | Reverse-scored; verify original Chinese wording | 0.805 |
| Reduced personal accomplishment | JB22 | I can appropriately handle problems encountered in fitness instruction. | Reverse-scored; PT-specific wording | 0.767 |

**C. Turnover intention**

| **Dimension** | **Code** | **English item wording** | **Adaptation/scoring information** | **Loading** |
| --- | --- | --- | --- | --- |
| Turnover intention | TI1 | I feel that my organization is not as good as I had imagined. | No further substantive modification documented | .786 |
| Turnover intention | TI2 | If I continue to stay with this organization, my future prospects may not be very good. | No further substantive modification documented | .798 |
| Turnover intention | TI3 | I may leave my current organization within the next one or two years. | No further substantive modification documented | .766 |
| Turnover intention | TI4 | If a suitable job becomes available, I would choose to leave. | No further substantive modification documented | .817 |
| Turnover intention | TI5 | I plan to leave within the next six months. | No further substantive modification documented | .800 |
